# Chromatin associations in *Arabidopsis* interphase nuclei

**DOI:** 10.3389/fgene.2014.00389

**Published:** 2014-11-13

**Authors:** Veit Schubert, Radoslaw Rudnik, Ingo Schubert

**Affiliations:** ^1^Leibniz Institute of Plant Genetics and Crop Plant Research (IPK) GaterslebenStadt Seeland, Germany; ^2^Faculty of Science and Central European Institute of Technology, Masaryk UniversityBrno, Czech Republic

**Keywords:** *Arabidopsis*, BAC, chromatin association, chromosome territory, expression, FISH, interphase nucleus, transcription

## Abstract

The arrangement of chromatin within interphase nuclei seems to be caused by topological constraints and related to gene expression depending on tissue and developmental stage. In yeast and animals it was found that homologous and heterologous chromatin association are required to realize faithful expression and DNA repair. To test whether such associations are present in plants we analyzed *Arabidopsis thaliana* interphase nuclei by FISH using probes from different chromosomes. We found that chromatin fiber movement and variable associations, although in general relatively seldom, may occur between euchromatin segments along chromosomes, sometimes even over large distances. The combination of euchromatin segments bearing high or low co-expressing genes did not reveal different association frequencies probably due to adjacent genes of deviating expression patterns. Based on previous data and on FISH analyses presented here, we conclude that the global interphase chromatin organization in *A. thaliana* is relatively stable, due to the location of its 10 centromeres at the nuclear periphery and of the telomeres mainly at the centrally localized nucleolus. Nevertheless, chromatin movement enables a flexible spatial genome arrangement in plant nuclei.

## Introduction

Interphase chromatin organization in relation to gene regulation and other nuclear functions is currently under intensive research (Bickmore and van Steensel, 2013). Spatial chromatin arrangement proves to be surprisingly stable throughout mitotic cycles (Gerlich et al., 2003; Walter et al., 2003; Berr and Schubert, 2007). Nevertheless, chromatin fiber motility and changing associations are the prerequisite for chromatin interactions important for regulating gene expression, DNA replication and repair (Fraser and Bickmore, 2007; Dekker, 2008; Zhang et al., 2012). Such interactions are not random. They may occur intra-chromosomally between chromatin regions that are close together on a linear chromosome (short-range interactions) or between chromatin regions far from each other on the same chromosome (long-range interactions), or on different chromosomes (inter-chromosomal) (Cope et al., 2010; Woodcock and Ghosh, 2010; Feng et al., 2014).

The occurrence of spatial associations between chromatin segments were proven for centromeres, telomeres, replication origins, enhancers, promoters and chromosome break ends (Cavalli, 2007; Duan et al., 2010; Obe and Durante, 2010; Li et al., 2012; Sanyal et al., 2012; Crevillen et al., 2013; Dekker et al., 2013; Jin et al., 2013).

Furthermore, in yeast, Drosophila and mammals interaction has been shown for highly co-expressed genes (Osborne et al., 2004, 2007; Brown et al., 2006, 2008; Tanizawa et al., 2010; Gibcus and Dekker, 2012, 2013; Hou and Corces, 2012). The interplay between spatial genome organization and gene activity seems to be realized by a probabilistic self-organizing and self-perpetuating system based on epigenetic dynamics (Cavalli and Misteli, 2013; Voss and Hager, 2014).

Mammalian genomes are organized in topological domains flanked by CTCF (Master Genome Regulator Protein CCCTC-binding Factor) insulator protein-binding sites. Such domains, decondensed by RNA polymerase and topoisomerase, comprise transcriptionally active “open chromatin” fibers (Dixon et al., 2012; Naughton et al., 2013). CTCF and the SMC (Structural Maintenance of Chromosome) complexes (cohesin, condensin and the SMC5/6 complex) facilitate spatial association between distant DNA elements. Such transient associations are important for regulating transcription and repair, respectively (Parelho et al., 2008; Watanabe et al., 2009; Carretero et al., 2010; Ohlsson et al., 2010; Boyle et al., 2011; Poon and Mekhail, 2011; Sofueva and Hadjur, 2012; Yang and Corces, 2012; Huang et al., 2013).

To track chromatin fiber movements in living cells, fluorescent reporter proteins can be applied (Robinett et al., 1996; Marshall et al., 1997; Kato and Lam, 2001; Vazquez et al., 2001; Matzke et al., 2003, 2005; Rosin et al., 2008; Rosa et al., 2013). In fixed tissues single chromatin associations can be analyzed by applying specific DNA and RNA probes for FISH (Fransz et al., 2002; Branco and Pombo, 2006; Shopland et al., 2006; Schubert and Shaw, 2011; Schubert et al., 2012). Even chromatin interactions along the entire genome can be studied using the chromatin proximity-ligation assay “chromosome conformation capture” (3C) and its derivatives (Simonis et al., 2006; Dostie and Dekker, 2007; de Wit and de Laat, 2012; Dekker et al., 2013).

The latter methods were applied to *A. thaliana* nuclei which contain, compared to animals, relatively small interactive regions of similar epigenetic features throughout the genome. Increased interaction frequencies were proven between subtelomeric and pericentromeric regions (Moissiard et al., 2012; Grob et al., 2013; Feng et al., 2014).

These results correspond to cytological observations that, in addition to the mainly random but stable distribution of distinct chromosome territories (CTs) in interphase nuclei, (peri)centromeres and (sub)telomeres tend to associate (Armstrong et al., 2001; Fransz et al., 2002; Pecinka et al., 2004; Schubert et al., 2006, 2012).

Here we show, based on DNA-FISH, that in *A. thaliana* leaf cell interphase nuclei, in addition to a stable global interphase chromatin organization, intra- and inter-chromosomal associations appear, albeit at a low frequency. In addition, we found that interstitial euchromatin segments containing highly co-expressing genes do not consistently associate more often than those containing low co-expressing genes, possibly due to adjacent genes showing a lower expression.

## Materials and methods

### Preparation of nuclei, probe labeling and fish

*A. thaliana* (L.) Heynh. (Columbia) plants were grown under short-day conditions (8-h light/16-h dark) at 21°C. Then nuclei from differentiated cells were isolated from rosette leaves and flow-sorted after formaldehyde fixation using a FACS Aria (BD Biosciences) according to their ploidy level as described by Pecinka et al. (2004). Similarly, root cell nuclei were isolated and sorted from 3d-old seedlings.

The *A. thaliana* BACs were obtained from the *Arabidopsis* Biological Resource Center (Columbus, OH, USA). To analyse euchromatin segments of chromosomes 1, 3, and 5, single BACs and BAC contigs were labeled by nick translation, either individually or, for painting of segments >100 kb, arranged into pools (4 or 5 BACs each) (Pecinka et al., 2004) with TexasRed-dUTP, Alexa488-dUTP and Cy3-dUTP according to Ward (2002). CT1top was labeled with BACs T25K16-F12K21, CT1bottom with F2J6-F23A5, CT5bottom with F5H8-K9I9, the ~2.8 and 2.6 Mb segments at mid-arm position of CT1top with F11A6-F5A9 and T10O24-F28G4, respectively, and the ~760 kb contig at CT1bottom arm with BACs F8A5-F19K23. The single BACs used for painting of these chromosome arms and segments are listed in Table S1.

FISH was performed according to Schubert et al. (2001). Nuclei were counterstained with DAPI (1 μg/ml) in Vectashield (Vector Laboratories).

### Microscopic evaluation, image processing and statistics

Analysis of FISH signals was performed with an epifluorescence microscope (Zeiss Axiophot) using a 100x/1.45 Zeiss α plan-fluar objective and a 3-chip Sony (DXC-950P) color camera. Images were captured separately for each fluorochrome using appropriate excitation and emission filters. Images were merged using Adobe Photoshop 6.0 software (Adobe Systems, San Jose, USA).

Differential Interference Contrast (DIC) Microscopy was performed with the same microscope using a 63x/1.40 Zeiss DIC objective and the DOM (Digital Optical Microscope) software (Schwertner, Jena) to acquire time-lapse movies from stamen hair cells of *Tradescantia paludosa* E. S. Anderson and Woodson.

The frequencies of heterologous *cis* and *trans* associations (% associated FISH signals) between interstitial euchromatin segments on chromosomes 1, 3, and 5 in comparison to the randomly expected values of the Random Spatial Distribution (RSD) model were compared for 2C nuclei by the two-sided Fisher's exact test. The RSD model simulates round-shaped homologous and heterologous chromosome segments (corresponding to BAC signal areas) with coordinates determined randomly in a virtual interphase nucleus. The frequency of attachment and overlapping of two BAC areas, taken as homologous or heterologous association, is considered to be random (Schubert et al., 2012).

### *In silico* and co-response expression analyses

The genes on the BACs of interest were identified using the Arabidopsis.org database. Due to requirements regarding the input parameters, only genes without splice variants were used. Conditional pair-wise gene-to-gene co-response queries for single genes (sGQ) and multiple genes (mGQ) were generated using the Comprehensive Systems Biology database (CSB.DB; Steinhauser et al., 2004). Since the entry mask within the database allows no input of punctuation marks regarding the gene ID only genes without splice variants were used for analysis. The co-expression values were generated by Spearman's non-parametric measure of correlation (rs). A value of +1 means a perfect positive correlation while a value of −1 represents a perfect negative correlation. The *p*-value describes the probability of the correlation coefficient and ranges from zero to one. In case of a small *p*-value, the observed correlation is likely to be not random.

Expression analysis for selected genes from the fourth rosette leaf (ATGE_13) of 17 days-old Columbia wild-type plants was performed using the AtGenExpress Developmental series database. Resulting values are log2 mean-normalized microarray signal intensities (Schmid et al., 2005).

## Results

We applied BACs and BAC contigs containing *A. thaliana* euchromatin segments labeled in different colors for FISH experiments on flow sorted differentiated leaf nuclei to elucidate the degree of homologous and heterologous chromatin associations.

### CTs are mostly compact but can also decondense

**Figure 1:**
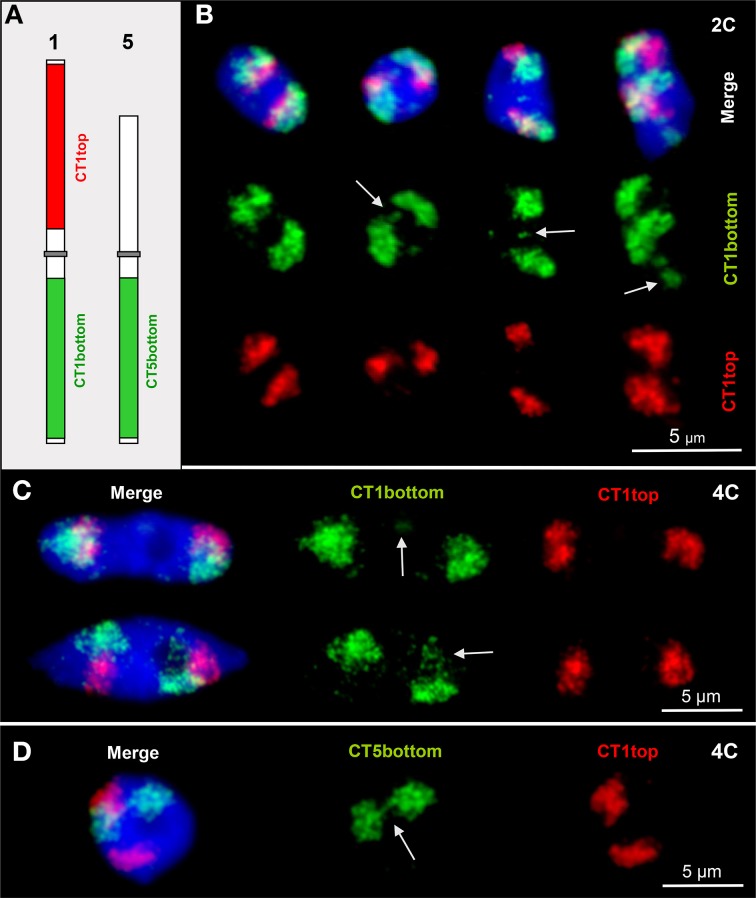
**CTs are mainly compact but allow relaxation, chromatin fiber out looping and association. (A)** Scheme of chromosomes 1 and 5 showing the labeling of the euchromatic CT1top, CT1bottom and CT5bottom arm segments by FISH. **(B)** Arrows mark chromatin domains clearly located outside of their CTs in 2C nuclei. The left nucleus contains compact CTs of both homologs without external chromatin. **(C)** Minor (top arrow) and larger (bottom arrow) chromatin segments separated from CT1bottom arms in 4C nuclei. **(D)** Due to chromatin fiber out looping in this 4C nucleus, the homologous CT5bottom arms are connected (arrow).

*A. thaliana* chromosomes are organized in distinct CTs which are only seldom completely dispersed (Pecinka et al., 2004; Schubert et al., 2012). Here we show that after labeling CT1top, CT1bottom and CT5bottom in different colors by FISH also small or larger chromatin segments may be localized distantly apart from their compact CTs. Such chromatin fiber movements may also lead (once in 300 nuclei analyzed) to chromatin association between distantly located CTs by out-looping from their own CT (Figure 1).

### Euchromatin segments may loop out apart from their CTs

**Figure 2:**
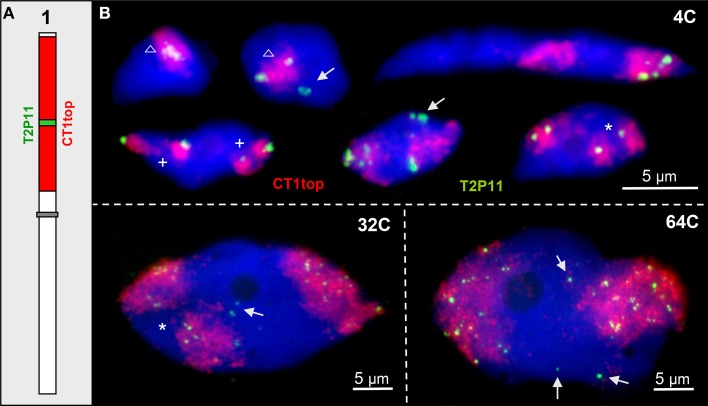
**~85 kb euchromatin segments may loop out from their CTs**. **(A)** Scheme of chromosome 1 showing the labeling of CT1top arm, and BAC T2P11 therein in different color as used for FISH. **(B)** Examples of chromatin configurations in 4C, 32C, and 64C nuclei. Sister chromatid territories of one (asterisks) or both (cross) homologs may be separated due to missing chromosome arm cohesion. The T2P11 segment is associated with (triangle) or separated from its CT (arrows). The strongly elongated 4C nucleus shows the T2P11 segment absent from the left CT and moved into the homologous right CT.

In ~13% of 4C nuclei an interstitial ~85 kb euchromatin segment cloned in BAC T2P11 was localized outside of its CT (Pecinka et al., 2004). This segment may also loop out in nuclei of higher ploidy levels of 32C and 64C. In addition, due to chromatin fiber elongation and distant movements the segment may appear aside from its CT while the homologous segment is present in its CT (once observed in 150 nuclei analyzed) (Figure 2).

### Distant interstitial chromatin associations appear rarely

**Figure 3:**
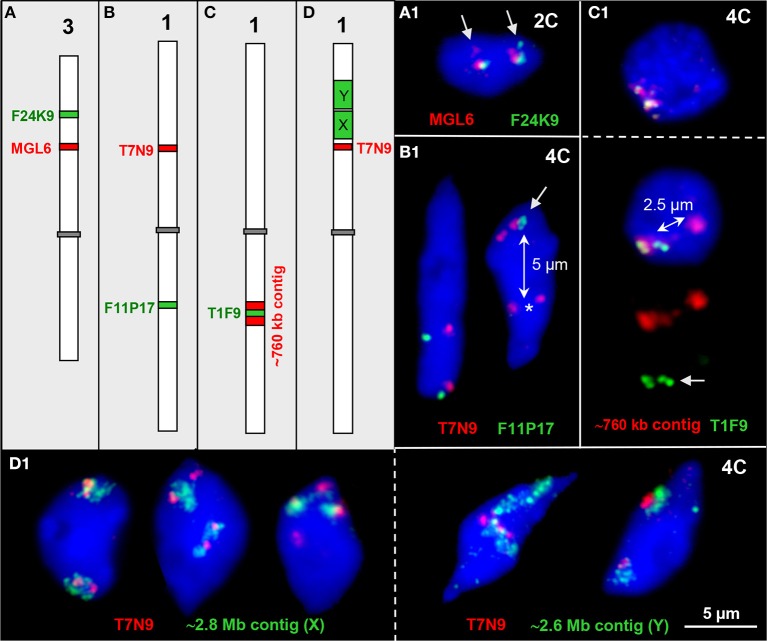
**Interstitial chromatin associations. (A–D)** Schemes of chromosomes 1 and 3 showing interstitial positions of single BAC and BAC contig inserts in different color as used for FISH. **(A1)** Parts of both ~100 kb euchromatin segments are elongated in one homolog each thus showing a second signal (arrows) in an 2C nucleus. **(B1)** The ~100 kb segments T7N9 and F11P17 located on different arms of chromosome 1 may both be cohesive (left) or one may be separated (right, T7N9) in 4C nuclei. In the right nucleus all four F11P17 sister chromatid segments are cohesive and associated (arrow) indicated by the location apart (~5 μm, double arrow) from the lower located homolog position marked by the two T7N9 signals (asterisk). **(C1)** The top 4C nucleus contains the 760 kb contig of both homologs in close vicinity. The bottom nucleus displays the same contig, but the T1F9 segment of one homolog moved apart from its contig position (~2.5 μm, double arrow) toward the second homolog (arrow). **(D1)** Examples of chromatin configurations in 4C nuclei labeled by ~2.8 Mb (X) and ~2.6 Mb BAC (Y) contigs, respectively, in combination with the segment T7N9 from the same chromosome arm. In the first nucleus the green contigs are associated with the T7N9 segment, in the second nucleus the segments are located at the edges of the contigs, and in the third nucleus two of the non-cohesive T7N9 segments are present distantly from the contigs. The fourth and fifth nuclei demonstrate, that the T7N9 segments, although located ~3.3 Mb apart from the ~2.6 Mb contig may be localized within this domain.

For testing to what degree homologous and heterologous chromatin associations appear between distinct chromatin segments of different genetic positions, we labeled euchromatin segments of different sizes (~80 kb, ~760 kb, ~2.6 Mb, ~2.8 Mb) in different colors and combined them for FISH (Figure 3).

Two ~100 kb segments on chromosome 3top may be associated, and a part of the segment can be located distantly (1.0%, *n* = 150) in 2C nuclei, obviously due to chromatin elongation (Figure 3A1).

The ~100 kb segments T7N9 and F11P17 at top and bottom mid-arm positions of chromosome 1, respectively, were mainly localized in close proximity because apparently the centromere keeps both arm CTs together (Figures 1B,C; Pecinka et al., 2004). However, in one out of 300 nuclei the segment F11P17 associates with its homologous counterpart distantly apart from its own CT (Figure 3B1).

Figure 3C1 demonstrates that the euchromatin segment T1F9 as a central part of a ~760 kb segment from bottom arm of chromosome 1 can move, but very seldom (observed in only one out of 250 nuclei) ~2.5 μm apart from its CT toward the second homolog.

Vice versa we observed that at mid-arm positions of chromosome arm 1top an ~80 kb segment ~0.5 and 3.3 Mb apart from the ~2.8 and 2.6 Mb segments, respectively, may become localized either apart (2.7%, *n* = 150) or within (4.0%, *n* = 150) the larger subdomains (Figure 3D1).

### The degree of condensation and the frequency of co-localisation may differ for adjacent euchromatin segments

Previously we found that euchromatin segments along *A. thaliana* chromosomes may be variably condensed. An increased chromatin fiber elongation (more than two or four signals in 2C and 4C nuclei, respectively) was conspicuous at some subtelomeric and close to some pericentromeric positions (Schubert et al., 2012). Here we confirm the frequent occurrence of elongation for a subtelomeric segment of chromosome 3 cloned in BAC F16M2, by showing that it occurs with a frequency of 24.0% (*n* = 200) in 2C and 29.5% (*n* = 43) in 4C nuclei, respectively. In contrast, the adjacent segment T20O10 showed in the same 2C and 4C nuclei only 9.0 and 7.0% elongation demonstrating that the degree of chromatin condensation can vary within a ~160 kb segment.

**Figure 4:**
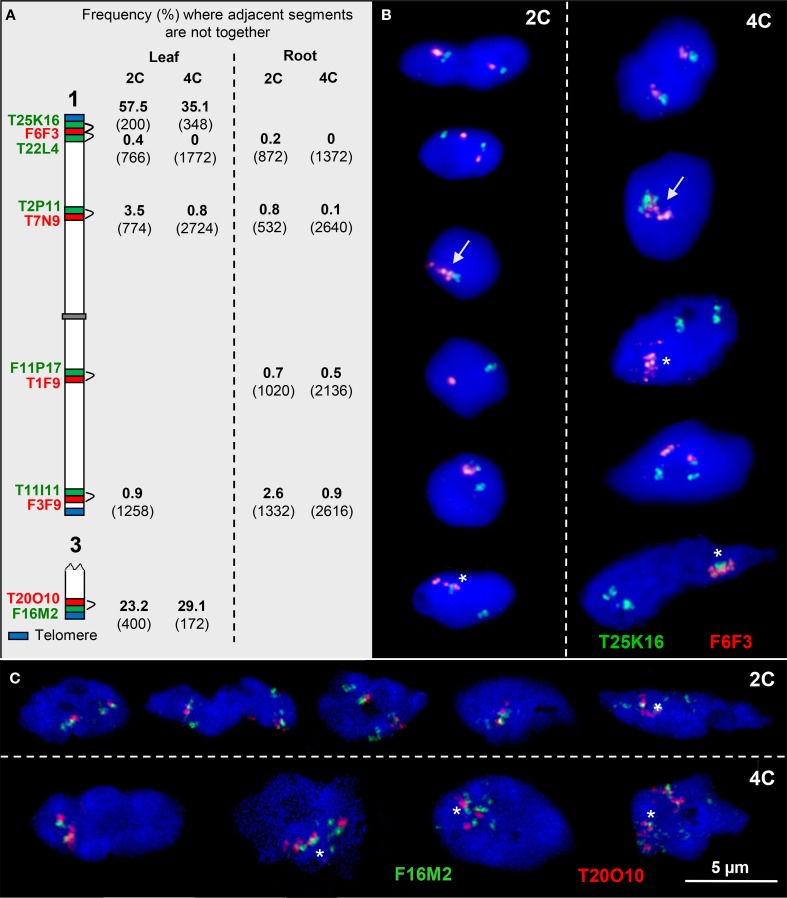
**Configurations of adjacent euchromatin segments. (A)** Scheme of chromosomes 1 and 3 showing the subtelomeric and interstitial position of adjacent segments (probed by two BACs in different colors). The percentage of nuclei where the adjacent segments were found not close together in 2C and 4C leaf and root nuclei, respectively, is indicated (number of investigated homologs (2C) and of sister chromatids (4C) in parentheses). **(B)** The top 2C and 4C nuclei show the T25K16 and F6F3 segments, as expected, close together. In the nuclei below, the homologous segments may also be completely (arrows) or partially associated (asterisks). In addition, due to chromatin fiber movement by elongation and/or sister chromatid separation, the adjacent segments may be localized distantly from each other. **(C)** Similar configurations as in **(B)**. In particular segment F16M2 appears frequently elongated.

To test whether adjacent euchromatin segments along chromosome arms may loop out compared to each other, we tested 2C and 4C leaf and root nuclei with two overlapping BACs in different color by FISH. The frequency at which such pairs of BACs were not close together was relatively low along the chromosome arms (0–3.5%) in root and in leaf nuclei. However, at the subtelomeres of chromosome 1top and chromosome 3bottom arms the frequency of positional separation of the corresponding BAC pairs was clearly increased (up to 57.5% in 2C and 35.1% in 4C leaf nuclei) (Figure 4).

The mean cohesion frequencies per homolog in 4C leaf nuclei of 58.1% for T20O10 and 25.6% for the adjacent F16M2 (*n* = 86) suggest an increase of sister chromatid separation below the chromosome termini.

In summary, from previous (Schubert et al., 2012) and these results we conclude that at subtelomeres and around the pericentromeres, due to low chromatin condensation and decreased sister chromatid cohesion compared to other regions, there is a higher chromatin flexibility to achieve homologous and heterologous associations.

### The association frequency of chromatin segments containing high or low co-expressing genes is globally not clearly different within a specific tissue

**Figure 5:**
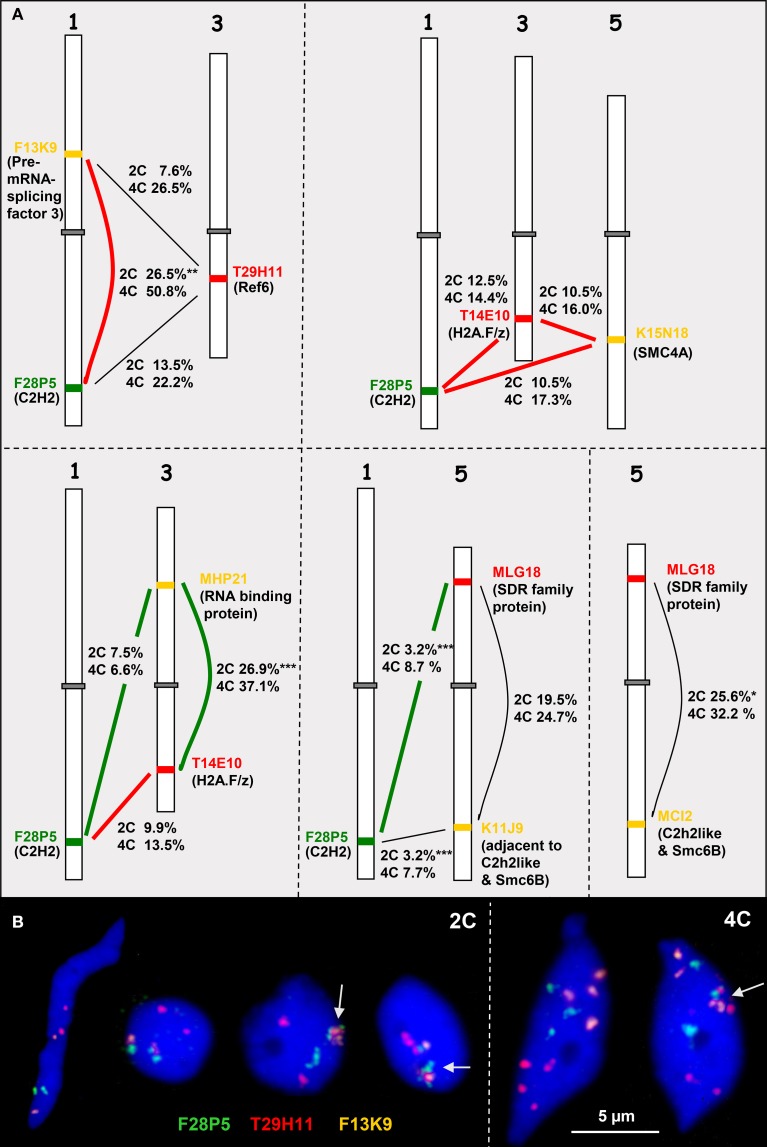
**Heterologous associations in cis and trans**. **(A)** Schemes of chromosomes 1, 3, and 5 showing euchromatin segments (probed by BACs) containing high (connected by bold red lines) or low (bold green lines) co-expressing genes (encoded proteins in parentheses). The percentage of association in *cis* or *trans* in 2C and 4C nuclei is indicated (for number of nuclei analyzed see Table S3). The thin black lines indicate combinations with missing co-expression data. **(B)** Examples of 2C and 4C nuclei showing different configurations of euchromatic segments probes by BACS F28P5, T29H11, and F13K9. The segments may be associated (arrows) or separated. Compare 2C values with the simulated random values according to the RSD model for loci at different arms of the same chromosome (17.2%) and for loci located at different chromosomes (9.9%) (^*^P < 0.05, ^**^P < 0.01; ^***^P < 0.001).

To test whether euchromatin segments of *A. thaliana* bearing high or low co-expressing genes show a different association frequency, we performed FISH experiments with suitable BAC combinations as probes and determined their association frequency. The selected BACs contain genes coding for the SMC complex subunit SMC4A (a condensin subunit; Schubert, 2009) and the potential CTCF insulator factors C2H2 and REF6 (www.arabidopsis.org) which are known to act in a concerted manner and to be involved in chromatin organization and transcriptional regulation (Poon and Mekhail, 2011; Huang et al., 2013). With these genes as reference (except Ref6 because data were not available) highly and lowly co-expressing genes were determined based on the Comprehensive Systems Biology database (CSB.DB; Steinhauser et al., 2004) (Table S2). Then the BACs bearing these genes were labeled in different colors and hybridized in five different combinations of probes from chromosomes 1, 3, and 5 in *cis* or *trans* to 2C and 4C leaf nuclei (Figure 5).

In addition to actively induced association, random association of chromatin segments may appear and can be calculated based on the RSD model. In 2C *A. thaliana* nuclei the frequency of random associations between segments located on different arms of the same chromosome (*cis*) and between segments on different chromosomes (*trans*) accounts to 17.2 and 9.9%, respectively (Schubert et al., 2012).

When compared to the random values it becomes obvious that significant deviations in both directions occur but are not consistently correlated with BACs containing either high co-expressing genes or those containing low co-expressing genes. BAC combinations in *cis* and *trans* may show similar deviations from the randomly expected values, e.g., only the *cis* association values of the BAC combination F13K9-F28P5 containing the highly co-expressing genes coding for the Pre-mRNA-splicing factor 3 and C2H2 proteins were clearly increased in 2C and 4C nuclei. However, similarly frequent association in *cis* was also found for the BACs MHP21 and T14E10 bearing low co-expressing genes on different arms of chromosome 3 (Figure 5A, Table S3).

The parts of the BACs comprising the genes that were compared for expression are of different size (~6 kb up to ~127 kb) and contain different numbers of genes (3 up to 36). The expression values for these genes in rosette leaves (AtGenExpress Developmental series database) were used to calculate the mean expression degree per BAC segment (Table S4). Fifteen genes on BAC F28P5 show together after normalization a relatively high mean expression value (~2.34) but with a high variability (*s* = 6.55). This segment associates in *trans* with the segment T29H11 (mean expression 0.40) in 13.5% (2C) and 22.2% (4C) of nuclei. However, the *trans* association frequency of F28P5 with K11J9 (mean expression 1.86) is only 3.2% in 2C and 7.7% in 4C nuclei. The mean expression value (0.57) of segment F13K9 is relatively low but the *cis* association with F28P5 is significantly more frequent than expected at random. Thus, it seems that there is no correlation between association frequency of interstitial euchromatin segments in *cis* or *trans* and the mean degree of gene expression in these segments.

In summary, we conclude that in general interstitial euchromatin segments bearing high or low expressing genes do not reveal different association frequencies probably due to adjacent genes of deviating expression patterns. The possibility, that single high or low expressing genes exhibit different association frequencies could be tested in a laborious program in future using ~3 kb large gene-containing FISH probes.

### Life-cell imaging confirms the relative stable interphase chromatin organization of plant nuclei

**Figure 6:**
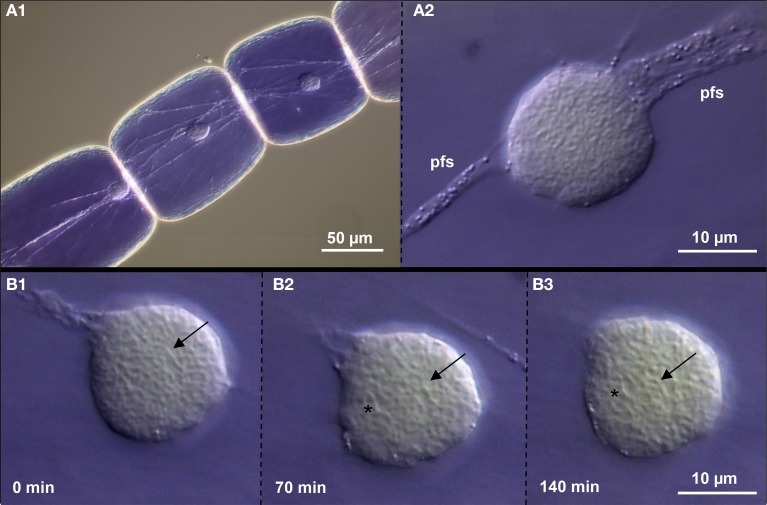
**Chromatin dynamics in living *Tradescantia paludosa* nuclei**. **(A)** The position of nuclei in stamen hair cells **(A1)** is kept by attached plasma fiber strands (pfs) **(A2)**. **(B)** Compared to the starting point of time **(B1)** the chromatin fibers mainly keep their position (arrows) after 70 min **(B2)** and 140 min **(B3)**. But in addition, other vesicular structures (asterisks) appear.

We used DIC microscopy to analyse the dynamics of chromatin in interphase nuclei of *Tradescantia* stamen hairs. During 120 min we observed that the nuclei attached to plasma fibers move and change their shape only slightly. Also the chromatin fibers inside keep mainly their position. But in addition, other vesicular structures (possibly nuclear bodies containing components involved in splicing, transcription or gene silencing; Del Prete et al., 2014) appear (Figure 6; Suppl. movie 1). Thus, we confirm also in living tissue that plant interphase nuclei maintain their global chromatin organization for longer times in spite of showing some flexibility.

## Discussion

### Despite a generally stable chromatin arrangement, variable intra- and interchromosomal associations occur in plant nuclei

The similar 3D organization of nuclei from diverse mammalian cell populations (Lieberman-Aiden et al., 2009; Dixon et al., 2012; Zhang et al., 2012) suggests a fundamental state of chromatin arrangement which enables essential functions during development and in response to environmental changes (Bickmore and van Steensel, 2013). The random Brownian motion of chromatin in *Drosophila* interphase nuclei was shown to be constrained within a limited nuclear sub-region but during the cell cycle motions over long distances appear (Vazquez et al., 2001). Constrained chromatin mobility is also present in *A. thaliana* nuclei. The constrained area increases with the level of endopolyploidy (Kato and Lam, 2003; Rosin et al., 2008), possibly due to a decreased cohesion of the chromatids within their CTs (Schubert et al., 2012). Adjacent replicated chromatin segments can be cohesive or separated within a Mb range in differentiated nuclei. In rare cases the minimum extension of cohesive sites as well as of distances between them may fall below ~500 kb (Schubert et al., 2008) allowing the movement of short chromatin segments.

Here we demonstrate restricted long-term chromatin motility also for living *Tradescantia* nuclei. Nevertheless, positional re-orientation may occur during plant development and in response to environmental stress, possibly to facilitate transcriptional reprogramming. For instance, heterochromatin reorganization in *A. thaliana* nuclei was observed during megaspore mother cell and gamete formation (Baroux et al., 2011; She et al., 2013), during endosperm development (Baroux et al., 2007), seed maturation (van Zanten et al., 2011, 2012a), early after germination (Mathieu et al., 2003) and after light exposure (Tessadori et al., 2007, 2009; van Zanten et al., 2010a,b, 2012b).

In spite of a generally stable spatial chromatin arrangement, euchromatin fiber movements may occur, sometimes even over large distances, as we have proven by FISH. Compared to interstitial positions it seems that especially subtelomeres and pericentromeres display a higher chromatin flexibility. Our data are in agreement with the findings achieved by chromatin proximity-ligation assays showing that increased interaction frequencies are present between subtelomeric, but also pericentromeric regions (Moissiard et al., 2012; Grob et al., 2013; Feng et al., 2014).

The low frequency (~1% of nuclei), at which adjacent interstitial chromatin segments are not located together may come from analyzing a differentiated leaf cell population comprising nuclei of different transcriptional state (out-looping of transcribed regions). Thus, essential chromatin fiber movements within nuclei might be restricted to a few nuclei and/or last a very short time only.

### Expression does not influence the global chromatin organization

FISH analysis (Shopland et al., 2006) and chromatin proximity-ligation assays demonstrated for animals that active gene-dense domains associate with each other mainly in *cis* but also in *trans* (Simonis et al., 2006; Lieberman-Aiden et al., 2009; Hakim et al., 2011; Yaffe and Tanay, 2011; Hou et al., 2012; Kalhor et al., 2012; Sexton et al., 2012; Zhang et al., 2012). Branco and Pombo (2006) visualized by FISH that the activated human histocompatibility complex may move into the CTs of other chromosomes. In contrast, Palstra et al. (2008) suggest that the binding of specific trans-acting factors and the arrangement and epigenetic modification of nucleosomes along the DNA fiber influence long-range chromatin associations more than active transcription. In human fibroblasts the radial chromatin arrangement (gene-dense, transcriptionally active chromatin preferentially in the nuclear interior and gene-poor chromatin at the nuclear envelope) is rather shaped by the local gene density than by gene expression (Küpper et al., 2007). A locus preferentially located outside of its own CT core may have an increased probability to interact with chromatin from other chromosomes, independent of its transcriptional activity (Bickmore and van Steensel, 2013).

In animals transcription is thought to proceed in distinct “transcription factories” (Chakalova et al., 2005; Chakalova and Fraser, 2010; Ferrai et al., 2010; Rieder et al., 2012; Papantonis and Cook, 2013) comprising ca. 4–30 RNA polymerase II (RNAPII) molecules (Iborra et al., 1996; Jackson et al., 1998; Martin and Pombo, 2003). In plants, RNAPII foci rather form an overall distributed network within euchromatin (Schubert, 2014) and transcription sites are distributed uniformly throughout the nucleoplasm (Abranches et al., 1998). Thus, compared to animals, less chromatin fiber movement should be necessary in plants for gene transcription. This idea is supported by the observation that epigenetic marks of active chromatin did not show a co-localization with highly associated chromatin segments and are also not present in pericentromeric regions that show the strongest interactions in the genome according to data found in Hi-C experiments. This suggests a lack of clustering of the most actively transcribed genes (Feng et al., 2014).

An *A. thaliana* chromatin segment containing 22 genes including the flowering locus FWA, which is constitutively expressed in leaf cells of *fwa* mutants showed an inconsistent increase of out-looping from its CT compared to wild-type. Seven of the other 21 genes of the fwa-containing segment are strongly transcribed in leaf cells; three of them within 20 kb upstream or downstream of fwa (http://www.weigelworld.org). It is not yet clear whether such a density of active genes is sufficient to be detected by FISH as a region looped out from its CT (Schubert et al., 2006; Schubert and Shaw, 2011).

Here we found for *A. thaliana* nuclei no differences in association frequencies between interstitial euchromatin segments bearing high or low expressing genes. On the other hand, Rosa et al. (2013) demonstrated in *A. thaliana* nuclei that gene positioning and transcriptional activity are linked through Polycomb-mediated epigenetic mechanisms in response to cold treatment. Schubert and Shaw (2011) found glutenin genes in wheat endosperm and Wegel et al. (2009) adjacent genes which are involved in the biosynthesis of the secondary metabolite avenacin of oat, to be decondensed on activation.

There is increasing proof from literature, that *A. thaliana* nuclei may undergo global chromatin rearrangement during development and in response to environmental changes (Baroux et al., 2007, 2011; Tessadori et al., 2007, 2009; van Zanten et al., 2010a,b, 2011, 2012a,b). Although, within nuclei of differentiated tissues the spatial chromatin arrangement is relatively stable, chromatin fiber movement, sometimes even over large distances, is possible, likely due to flexible chromatid cohesion, particularly in endopolyploid nuclei (Schubert et al., 2008, 2012). The more frequent intra- and interchromosomal associations at pericentromeres and at subtelomeres may be due to the localization and association of the centromeres at the nuclear periphery and of most of the telomeres around the nucleolus. The ability of heterochromatin dispersion at pericentromeres and the elongation of subtelomeric domains seem to allow an increased degree of homologous and heterologous interactions compared to interstitial euchromatin fibers. The difficulty to detect a correlation of high expression activity of euchromatin segments with *cis* and *trans* associations by FISH might be due to low expressing genes in close neighborhood and/or to interactions of short duration.

The improvement of our understanding of the interplay between spatial chromatin arrangement and distinct metabolic functions within nuclei is expected from (i) single cell analyses with fluorescent reporter proteins and new methods as genome editing (Wood et al., 2011), (ii) the application of artificial DNA-binding proteins to create new topological structures to study their functional consequences (Deng et al., 2012) as well as (iii) the manipulation of protein binding sites responsible for interactions between chromatin domains (Nora et al., 2012).

### Conflict of interest statement

The authors declare that the research was conducted in the absence of any commercial or financial relationships that could be construed as a potential conflict of interest.

## Supplementary material

Suppl. movie 1 **The behavior of a *Tradescantia paludosa* nucleus in a stamen hair cell during 120 min**.
