# Supplementary figures and images for: Chromatin associations in Arabidopsis interphase nuclei
Source: Front Genet. 2014 Nov 13;5:389. doi: 10.3389/fgene.2014.00389 (PMC4230181; doi:10.3389/fgene.2014.00389)

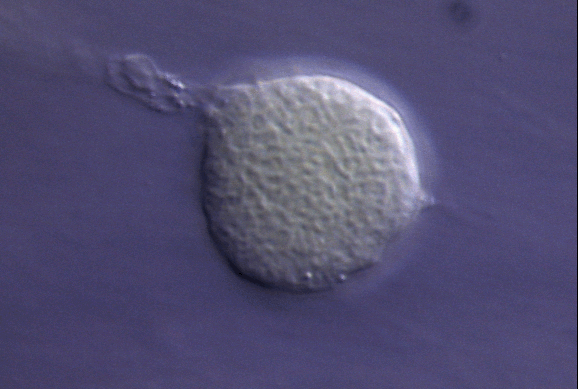

Supplement: Suppl. movie 1 — The behavior of a Tradescantia paludosa nucleus in a stamen hair cell during 120 min. [file Presentation1.ZIP › Chromatin-associations-in-Arabidopsis-Suppl-movie1.gif]
